# Time trends and geographical patterns in suicide among Greenland Inuit

**DOI:** 10.1186/s12888-023-04675-2

**Published:** 2023-03-21

**Authors:** Ivalu Katajavaara Seidler, Janne Schurmann Tolstrup, Peter Bjerregaard, Allison Crawford, Christina Viskum Lytken Larsen

**Affiliations:** 1 National Institute of Public Health, University of Southern Denmark, Copenhagen, Denmark; 2 Institute for Health and Nature, University of Greenland, Nuuk, Greenland; 3 Centre for Addiction and Mental Health, Toronto, Canada

**Keywords:** Suicide, Time trends, Inuit, Greenland, Register

## Abstract

**Background:**

Between 1980 and 2018 Greenland has had one of the highest suicide rates in the world with an average rate of 96 suicides per 100,000 people annually. The aim of this study is to investigate suicide rates in Greenland according to age, birth cohort, period, sex, place of residence and suicide method from 1970 until 2018.

**Methods:**

Suicide rates were examined using register and census data from 1970–2018 among Greenland Inuit. Rates were calculated by Poisson regression in Stata and by use of Excel. In analyses of the period trends, rates were standardized according to the World Standard Population 2000–2025.

**Results:**

The suicide rate has been declining since a peak at 120 suicides per 100,000 people annually in the 1980s but remained high at a rate of 81.3 suicides per 100,000 people annually from 2015–2018. Descriptive analyses point to the decrease in male suicides as the primary factor for the overall decreasing rates while the rate among women has been increasing. Simultaneously, the proportion of women who used a violent suicide method increased from 60% in 1970–1979 to 90% in 2010–2018. The highest rates are seen among young people, especially young men aged 20–24 years and youth suicide rates increased with later birth cohorts. When the rates started to increase in the 1980s both the capital Nuuk and East Greenland had the highest rates. Since then, the rate in Nuuk has declined while the rate in East Greenland was three times the national rate from 2015–2018.

**Conclusions:**

From 1970 to 1989 the suicide rate increased from 28.7 to 120.5 per 100,000 people mirroring a rapid societal transition in the post-colonial period. The rate has slowly declined from the peak in the 1980s but remains at a very high level. Young people in general are at risk, but the steady increase in the rate among women is worrying and there is a need to investigate underlying causes for this development.

**Supplementary Information:**

The online version contains supplementary material available at 10.1186/s12888-023-04675-2.

## Introduction

Greenland is the least densely populated country in the world with 56,000 people living scattered along the coast of the 2 million km^2^ island. The majority of the population are Inuit making up 90% of the population [1]. Inuit in Greenland are related to Inuit populations living across the Arctic sharing similar cultural heritages, language, but also challenges. Indigenous populations across the Arctic experience very high suicide rates compared to non-indigenous populations [2, 3]. These high rates are linked to colonization, rapid sociocultural and economic transformations leading to significant changes in lifestyles and livelihoods across a historically short span of 50 years [4–6]. This has been linked to the onset of events causing social problems, intergenerational traumas, high prevalence of adverse childhood experiences, and loss of cultural affiliation especially impacting youth mental health [5, 7, 8]. With one of the highest suicide rates in the world it is to great public health and societal value to continuously monitor suicide rates in regard of specific trends and risk factors [9]. Today Greenland experiences between 60 to 40 suicides every year and from 2000 to 2018 the average annual rate was 87.3 per 100,000 people [6, 10].

### A century of monitoring suicide in Greenland

The World Health Organization has stated that registration and ongoing monitoring of suicides are essential for national prevention strategies [9]. Registrations of suicides from 1901–1960 reported that suicide was a rare event in Greenland mainly related to psychiatric disease with rates varying from 0–3.5 suicides per 100,000 people annually [6, 11, 12]. After the initiation of an intensive modernization plan for Greenland in the 1950s the suicide rate increased dramatically from the 1960s and stabilized at a high level from 1980 and onwards with an average rate of 96 suicides per 100,000 people annually [6]. From 1968 until 2018, suicide has accounted for 10% of all deaths in Greenland contributing to a significantly lower life expectancy when comparing with Western countries and in the period of 2005–2009 suicide was the third leading single cause of death in Greenland [1, 5]. Methods of suicide are categorized as either violent or non-violent. Distinguishing the method of suicide is important because violent methods (e.g., hanging, shooting, or drowning) are associated with a higher lethality than non-violent methods [13]. The differences in lethality were investigated in a systematic review and meta-analyses that reported case fatality rates varying from 84.6 to 89.7% for violent methods such as hanging or suffocation and shooting to 8% for poisoning (non-violent method) [14]. There are several ways of defining which methods are violent or non-violent but a simple approach is to define non-violent methods as poisoning and all other methods as violent [13]. The type of method is associated with sex and the general pattern is that men use violent methods more often than women and thereby increasing the lethality [13, 14]. In Greenland research has found the same association between method and sex which may in turn explain some of the sex-related differences in suicide rates [15, 16].

### Sex, age, birth cohort and geography

Most suicides in Greenland are youth suicides with the highest rates among young men [6]. Regional differences have shifted across time and more recent research has found the highest rates in East and North Greenland as opposed to the capital Nuuk where rates have decreased from more than 150 suicides per 100,000 people annually in the 1980s to around 60 since the 2000s [6]. An inspection of birth cohorts show a pattern of higher rates of youth suicides in younger birth cohorts where the youngest birth cohorts include the age group 10–14 years [6]. Besides age, period and birth cohort are key indicators/variables to include when analyzing temporal trends in suicide rates because they reflect both historical context and the rapid sociocultural developments of the postcolonial period in Greenland dating from 1953 and forward [5–7, 17, 18].

### Objective

The study objective was to investigate suicide rates in Greenland according to age, birth cohort, period, sex, and place of residence from 1970 until 2018. To get a more detailed understanding of the main objective potential patterns in suicide methods according to sex during the study period were investigated. Results may offer important knowledge for future strategies and interventions to prevent suicides in Greenland.

## Methods

This study is a register-based study on the Greenland Inuit population from 1970 to 2018. Greenland Inuit here defined as both being born and living in Greenland. The exclusion of other ethnicities is because the majority of non-Greenlanders are Danes who have come to Greenland to work and who often stay for a limited time. These are assumed to have a significantly different risk profile for suicide, given that the suicide rate in Denmark is around seven times less than the rate Greenland based on rates from 2007 until 2018 [19].

### Data sources

We examined suicide rates using register and census data for Greenland Inuit. The Central Population Register covers all Greenlandic citizens each assigned with a unique personal identification number allowing for register-linkage. Information on suicide was obtained from the nationwide Greenland register of causes of death dating back to 1968. The register is updated regularly, and deaths are validated against the Central Population Register. From 1970 until 2018 1,952 suicides were registered among 19,766 deaths using the International Classification of Diseases ICD-8 and ICD-10 codes developed by the World Health Organization. Codes included for suicide were E950, E951, E953-E959 for ICD-8 and X60-X65, X70-X75, X78, X80, X83-X84 for ICD-10. All suicide deaths are verified by a physician who then completes a medical death certificate. The Danish Health Data Authority code deaths to ICD codes and the information is recorded in the Greenland register of causes of death. All deaths due to injuries require that the police are involved [20]. The Greenland causes of death register has been validated and compared to police records with good agreement on suicides. When comparing the death certificates with police records from 1977 to 1986, 370 of the 420 suicides from the death certificates were registered in the police records [21]. A more recent study evaluated the usability of the register to be of medium quality based on The Vital Statistics Performance Index for Quality [20]. Population size was based on information from the Central Population Register and obtained from the statistical databank of Statistics Greenland for 1977–2018 and combined with census data for 1970 and 1976 [10, 22, 23]. The data included 2,265,676 people annually from 1970 until 2018. We followed the RECORD applying for routinely collected data for reporting of the study [24] Since data are not publicly availably no specific coding of outcome has been provided in this paper.

### Method of suicide

Based on the ICD-8 and ICD-10 codes method of suicide was categorized into four categories: *poisoning*; *hanging*, *strangulation or suffocation*; *firearms or use of explosives* and *other*. The category *other* includes methods that were less prevalent such as *drowning, cutting or piecing instruments, jumping from high places, other and unspecified means, and late effects of self-inflicted injury*. Method-specific suicide rates were calculated on *hanging*, *strangulation or suffocation*, *firearms, or use of explosives* and *other*.

### Stratification variables

Sex was included to distinguish different rates in men and women. To display different risk at different ages across cohorts, ten-year birth cohorts were included from 1940 until 1999. To investigate geographical differences Greenland was divided into five regions based on socioeconomic conditions and remoteness from the capital (Table 1). Nuuk, the capital, is the largest and wealthiest community with the greatest variety of jobs, shops, and cultural institutions. The largest communities on the west coast, Maniitsoq and Sisimiut, are centrally located and in many ways intermediate between Nuuk and the more remote communities on the west coast, the latter offering fewer jobs, shops, and cultural institutions. The 50 smaller communities (often referred to as villages) on the west coast of Greenland offer few jobs, often only a single shop and rarely any cultural institutions. East Greenland is remote and suffers from a high unemployment rate and many social problems. In East Greenland there are 3,110 inhabitants living in two larger and five smaller communities. Smaller communities are generally defined as having less than 500 inhabitants [10].

**Table 1:** The five geographical regions used for stratification of suicide rates based on information from Statistics Greenland 2022 [10]

Region	Communities	Population size
Nuuk	The capital of Greenland	18,800 inhabitants
Central large communities on	The largest communities on the west coast, Maniitsoq and Sisimiut	8,108 inhabitants in total
Peripheral large communities in West Greenland	Larger communities often having more than 500 inhabitants in East Greenland	20,156 inhabitants in total
Small communities in West Greenland	Communities often having less than 500 inhabitants located in West Greenland	6,179 inhabitants in total
East Greenland	All communities in East Greenland	3,110 inhabitants in total

### Statistical analyses

Analyses were performed using the statistical software Stata version 17. Rates were calculated by Poisson regression in Stata and by use of Excel [25, 26]. The observations were assumed to be independent. To make rates comparable internationally the analyses of the period trends were standardized to the average of five year periods according to the World Standard Population 2000–2025 [27]. Differences in age distributions across time were taken into account in the analyses of suicide rates according to sex and period where rates were age adjusted according to the average age distribution of the Greenland population in period 2015–2018.

## Results

Table 2 shows the development of the suicide rate from 1970–2018. From 1970–1974 the rate was 29 per 100,000 people annually. Over a 15-year period the rate more than quadrupled peaking at 121 per 100,000 people annually in 1985–1989. The rate started declining from 1990 and in 2015–2018 the rate was 81 per 100,000 people annually. Among the 1,952 suicides registered from 1970–2018, 41 suicides were registered in children aged 11–14 years.

**Table 2:** Suicide and suicide rates from 1970 to 2018

Period	Suicides	Suicides/year	Population (risk time)	Suicide rate per 100,000 people annually	Period specific percent change	Standardized suicide rates per 100,000 people annually ^a^
1970–1974	56	11.2	195402	28.7	-	33.3
1975–1979	95	19.0	202427	46.9	63.8	47.8
1980–1984	210	42.0	210082	100.0	113.0	88.8
1985–1989	268	53.6	222348	120.5	20.6	99.7
1990–1994	240	48.0	236118	101.6	-15.7	96.9
1995–1999	251	50.2	245000	102.4	0.8	106.1
2000–2004	236	47.2	249123	94.7	-7.5	101.9
2005–2009	211	42.2	251990	83.7	-11.6	84.6
2010–2014	222	44.4	251744	88.2	5.3	86.7
2015–2018	163	32.6	200468	81.3	-7.8	80.4
Total	1952	40.7	2,264700	86.2	-	86.2

^a^Standardized according to World Standard Population 2000–2025

**Fig. 1:**
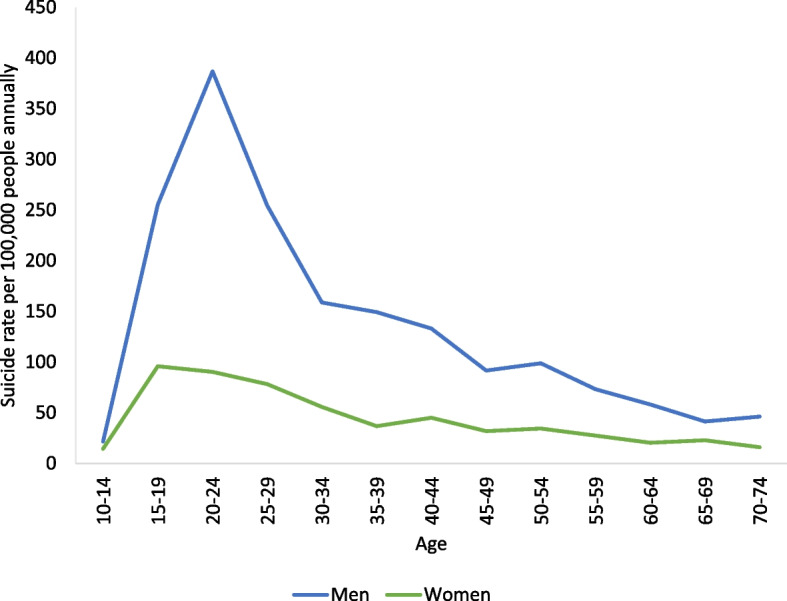
Age specific suicide rates by sex. Analysis of mortality data from 1970 to 2018

The highest rate was seen in young men aged 20–24 years with 387 suicides per 100,000 people annually followed by a decline with increasing age. Among women, young women aged 15–19 years had the highest rate of 96 suicides per 100,000 person years (Fig. 1).

**Fig. 2:**
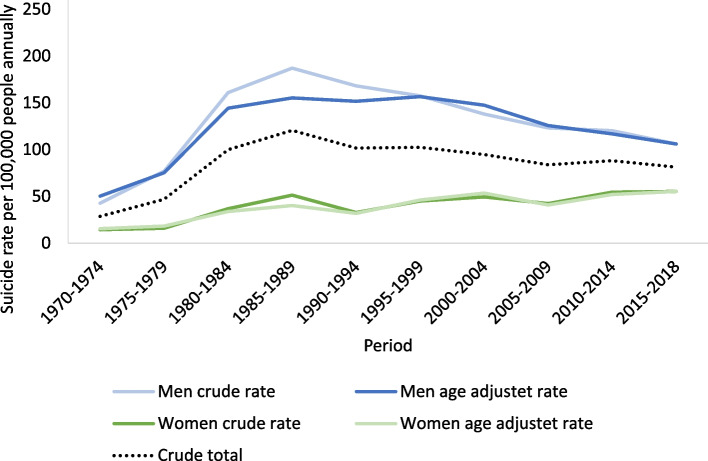
Period specific crude and age adjusted suicide rates by sex. Age adjusted rates were standardized according to the age distribution of 2015–2018. Analysis of mortality data from 1970 to 2018

**Fig. 3:**
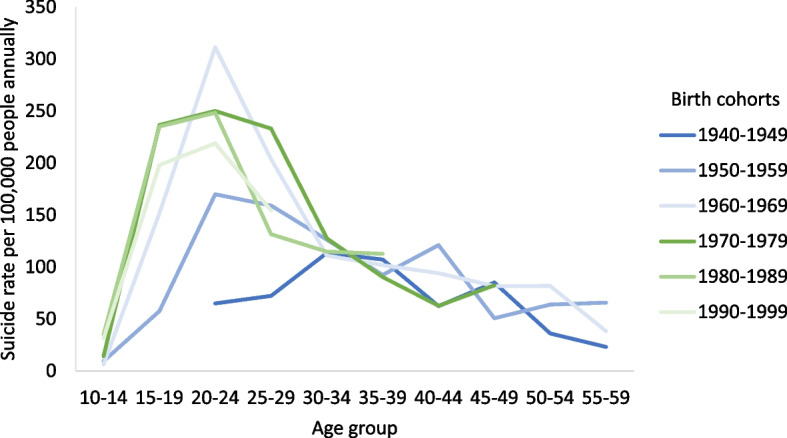
Age specific suicide rates by 10-year birth cohorts. Analysis of mortality data from 1970 to 2018

**Fig. 4:**
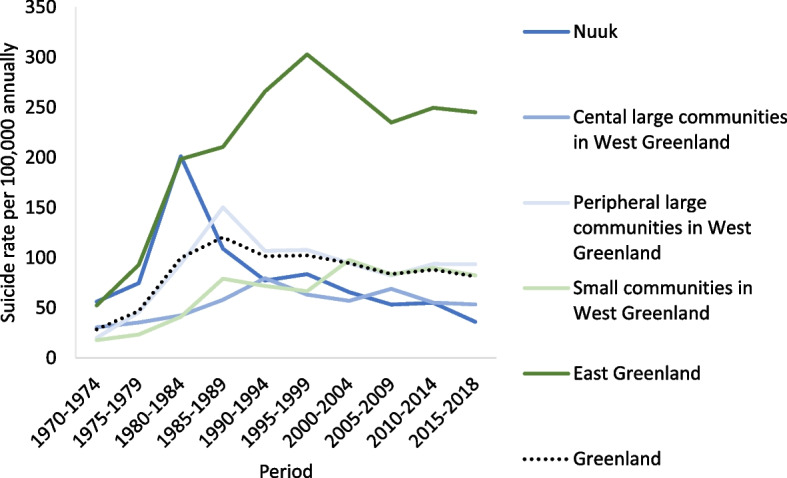
Period specific suicide rates by community size and geographical location. Analysis of mortality data from 1970 to 2018

The rates for both men and women increased until 1985–1989 whereafter the male rates declined while the female rates increased slowly until 2018 thus reducing the gender gap (Fig. 2). Figure 3 shows that birth cohorts born after 1960 had the highest suicide rates among the youngest age groups and that youth suicide rates continued to increase with later birth cohorts. The pattern was similar in the youngest birth cohort born in 1990–1999 but at a lower level compared to previous cohorts born between 1960 and 1989. Regional temporal trends are shown in Fig. 4. East Greenland experienced the highest rate of around 300 suicides per 100,000 people annually in 1995–1999. In the capital Nuuk, the rate was at its highest in the 1980s followed by a large decrease further increasing the differences in rates between West and East Greenland.

**Fig. 5:**
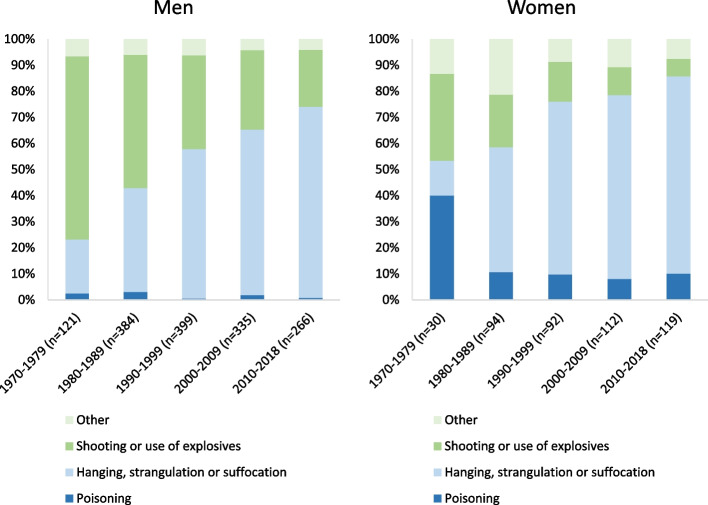
Method of suicide according to year in men and women. The category other includes drowning, cutting or piecing instruments, jumping from high places, other and unspecified means, and late effects of self-inflicted injury. Analysis of mortality data from 1970 to 2018

Figure 5 show that men mainly used violent methods for suicide with shooting or use of explosives as the most common methods in 1970–1979 (70%). Across time, suicide by hanging has become the most prevalent method in both sexes accounting for 73% of suicides in men and 76% in women from 2010–2018. Women had a high proportion of non-violent suicides (poisoning) at the beginning of the study period whereas the more violent methods, predominantly hanging, strangulation or suffocation became more prevalent during the study period increasing by 30%.

The method-specific suicide rate for hanging for both sexes increased from 7.3 per 100,000 people annually in the 1970s to 63 during 2010 to 2018. For shooting as method, the rate decreased from a peak of 49.7 per 100,000 people annually in the 1980s to 14.6 during 2010 to 2018. The rate of poisoning was 3.8 per 100,000 persons annually in the 1970s and after an increase to 5.1 in the 1980s it declined to 3.1 in the period from 2010 to 2018 (Table 3).

**Table 3:** Method-specific suicide rates. Analysis of mortality data from 1970 to 2018

Period	Other	Shooting or use of explosives	Hanging, strangulation or suffocation	Poisoning
1970–1979	3.0	23.9	7.3	3.8
1980–1989	9.9	49.7	45.8	5.1
1990–1999	6.9	32.6	60.2	2.3
2000–2009	5.2	22.7	58.2	3.0
2010–2018	4.4	14.6	63.0	3.1

## Discussion

The suicide rate in Greenland from 1970 until 2018 differed markedly according to age, birth cohort, period, sex, and place of residence. The suicide rate has been declining since a peak in the 1980s but remains high today as is the case among many other Indigenous populations compared to non-Indigenous populations [2]. Descriptive analyses point to the decrease in male suicides as the primary factor for the overall decreasing rates but the tendency of increasing rates in women is worrying. Simultaneously, the proportion of women who used a violent suicide method increased from 60% in 1970–1979 to 90% in 2010–2018 which in part explains the increase in the suicide rate among women. Overall hanging as a suicide specific method increased across the study period. This is the first study to identify a reducing gender gap in suicide rates among Greenlandic Inuit. As is the case across many Arctic Indigenous peoples the young men have had and continue to have the highest rates [6, 28, 29]. However, the authors have no clear explanation of why rates are decreasing in men and increasing in women.

### The epidemiology of suicide in Greenland

The findings of this paper are in line with previous epidemiological studies of suicides in Greenland. A systematic review that investigated epidemiological factors of suicide among young men in Greenland found the same increase in suicide rates as were shown in the analyses of period rates in the current study [30]. The results on suicide rates according to age and sex are in accordance with previous findings where youth aged 15 to 24 years have the highest rates across all age groups while young men have the highest rates overall [6, 31]. The same sex-related patterns are found among Inuit in Canada and Alaska and similar trends of a decrease in the rates have been shown [3].

#### Suicide methods

The documented high prevalence of hanging as suicide method is consistent with findings in a European context, where a high prevalence of hanging as suicide method has been found in both men (54.3%) and women (35.6%) [32]. Japan also struggle with high rates of youth suicides and the same pattern appears with hanging as the most prevalent method among adolescents, a prevalence that has increased since the 90 s in both sexes [33].

### Adverse childhood experiences

Research has pointed to the association of adverse childhood experiences (ACEs) and suicidality in Arctic Regions [28, 29, 34]. In Greenland ACEs have been linked to both suicides and suicidal thoughts [5–7, 35–38]. Experiencing alcohol problems often in the childhood home and sexual abuse was associated with up to four times higher odds of past year suicidal thoughts according to data from the population health surveys in Greenland [5, 17]. The latest survey data among youth aged 15–34 years reported that 41% of the young women were victims of sexual abuse before the age of 18, whereas the corresponding proportion was 12% in young men. The same survey found a cumulative effect between the number of experienced ACEs and prevalence of suicidal thoughts and attempts [39]. The high prevalence and the type of ACEs in Greenland may contribute to the pathogenesis of suicides: The high proportion of young women exposed to sexual abuse could be a contributing factor of the detected increase in female suicide rates. However, the population health surveys have shown a decrease in ACEs in the youngest birth cohorts compared to birth cohorts born in 1970–1989 where the proportion who experienced ACEs was very high [17]. The latter grew up during the period where the alcohol consumption was at its highest, which is paralleled in the high suicide rates of the birth cohorts. According to the figure, youth suicide began to increase in the generations born during the 1950s and more than quadrupled in the following 10-year birth cohorts after which youth suicide became the most prevalent across all age groups. Based on these observations there is a potential link between alcohol consumption, ACEs, and youth suicide.

### Intergenerational trauma

The trends in suicide rates of birth cohorts are also seen in other Arctic Indigenous peoples and has been interpreted as a sign of cultural cohort effects caused by cultural discontinuities related to colonialization and rapid societal changes [34, 40]. Problems with alcohol and ACEs does not only severely affect the individuals exposed to them but may have cumulative and long lasting consequences across generations [41]. Often these are referred to as intergenerational trauma. In the case of many Indigenous Peoples’, colonization of Indigenous land and lives, and rapid societal developments caused the onset of trauma and consequently intergenerational trauma [5, 7, 8, 42]. Intergenerational traumas are mirrored in the prevalence of both alcohol-related problems and ACEs. The prevalence has shifted across time and so has the impact on the generations exposed. Connection to culture has been identified as an important factor fostering resilience and improved mental health among those affected by intergenerational trauma [43–46]. The association between culture and resilience differs across generations: Older generations feel more connected to their cultural heritage enhancing the protective effect, whereas younger generations experience a more distorted cultural connection making them more vulnerable to the ongoing rapid societal transitions compared to the older generations [43].

### Geographical patterns of suicide

Whereas the rate in Nuuk started to decrease during the 1980s the rate in East Greenland continued to increase and stabilized at a very high level of three times the national rate. East Greenland is the most remote populated area of Greenland with less access to education, social- and health services, welfare, employment, and cultural institutions. A study on suicide in Greenland identified lack of access to education and employment as risk factors for suicide offering part of an explanation of the regional disparities in suicide rates [38]. Growing up in either a smaller community in West Greenland or in East Greenland is associated with a smaller likelihood of obtaining longer education which in turn affects the individual opportunities to fit into a modern society built on Western ideals [40, 47]. Living in smaller communities in West, East or North Greenland is further associated with lower income compared to the income of people living in the capital. The high suicide rate in East Greenland could further relate to the high prevalence of social problems and ACEs. In 2014 East Greenland had the highest prevalence of sexual abuse (46%), while violence and problems with alcohol consumption was most prevalent in Nuuk and other larger communities [47]. The Greenland Police releases a yearly report every year with statistics on ‘sexual relations with children under the age of 15’, and here East Greenland and larger peripheral communities have the highest numbers [48]. Survey data from 2018 among the Greenlandic youth aged 15–34 years support this clear geographical clustering of ACEs in the Eastern, Northern, and Southern municipalities. Nuuk (Sermersooq West) have the lowest prevalence but overall prevalence of alcohol in childhood home, violence in childhood home, and experience of sexual abuse before the age of 18 are high [39]. The geographical clustering of high suicide rates and related risk factors point to the need for different approaches in the preventive effort of suicide.

### Strengths and limitations

The included registries are regarded as complete and are updated annually. Using nationwide registers reduces the risk of selection bias while there are potential problems with information bias [49]. Problems with selection may occur due to migration, which is rather common in Greenland both with-in Greenland and often to Denmark where many people chose to settle down permanently or periodically as part of their educational path [1]. Determining whether this selection inherits bias will require more in-depth investigation of who migrates and their risk of suicide. Potential problems with misclassification of suicides as accidents and vice versa cannot be ruled out [21]. Since there are no knowledge on the direction or size of such it is not possible to determine the effect on the results of the study.

### Implications for prevention and health promotion

Temporal trends in the suicide rates are paralleled by trends in ACEs and can be linked to rapid societal developments in the post-colonial area [17, 50]. Research have found an association between ACEs and suicide attempts and ACEs are often reported as part of the background in suicide victims in Greenland [35–38, 51]. Based on this the prevention of ACEs would be an important factor in the preventive effort to reduce the number of suicides in Greenland. The regional differences in the suicide rate and prevalence of ACEs should be taken into consideration in the preventive efforts. Relevant preventive efforts and interventions to reduce ACEs should be focused on the capacity in the community. Community capacity cover empowerment of communities to provide healthy environments and to prevent and handle crises [52]. The findings of this paper underline the need to focus on suicide prevention among youth. Research have shown that there is a particular need to include a broad focus of not only the individual but also their surrounding community and relations [46]. Culture and relations have been found to enhance mental health by providing knowledge and connection to the culture, intergenerational relationships, and role models [45]. Suicide prevention interventions among Indigenous youth incorporating cultural aspects have been found to enhance the level of mental health [53, 54]. Arctic research is developing a more holistic strength-based approach to suicide prevention focusing on community strengths and protective factors fostering thriving communities [44, 45, 55–57]. Prevention should be culturally relevant, community-based, and include individual, family, social, historical, cultural, and environmental factors that constitute the base of the ‘iceberg’ of which suicide would figure as part of the visible tip above water [58]. In Greenland, land-based and intergenerational interventions for mental health among youth are being conducted and monitored. The Greenlandic Government is currently working on a new suicide prevention strategy expected to launch in 2023. The strategy includes perspectives from youth collected by the National Advocacy Center working for Childrens Rights, MIO [59].

## Conclusion

Across a 50-year period the suicide rate has increased drastically in Greenland mirroring a rapid societal transition in the post-colonial period. The rate has slowly declined from the peak in the 1980s but remains at a very high level. Young people, and especially young men, are at risk but the steady increase in the rate in women is worrying. This can in part be explained by the fact that there has been an increase in the proportion of violent suicide methods among women increasing the lethality. Research points to the importance of ACEs in relation to suicide risk and the high prevalences of ACEs among women may contribute to the decreasing gender gap but there is a need to investigate this further. Youth suicide increased with later birth cohorts born after 1949. The youngest birth cohort still exhibited the pattern of high youth suicide rates but at a lower level compared to older generations. These findings coincide with a decrease in ACEs and urbanization which authors believe is reflected in the rates overall and the development of geographical clustering of the highest rates. These factors must be taken into consideration in the preventive work along with a strong focus on the community and protective factors that increases intergenerational bonds and cultural connectedness. Factors which may heal intergenerational trauma and enhance a feeling of cultural identity creating a strong foundation for youth and foster thriving communities.

## Supplementary Information

**Additional file 1.**

## Data Availability

Data on population size are available from Statistics Greenland homepage, https://bank.stat.gl/pxweb/en/Greenland/. The Greenland register of causes of death is not publicly available. Researchers can apply for access to anonymized data via The Chief Medical Office: https://nun.gl/.

## Abbreviations

ACEs: Adverse childhood experiences
ICD: International Classification of Diseases

## References

[1] Statistics Greenland (2020). Greenland in figures 2020.

[2] Pollock NJ, Naicker K, Loro A, Mulay S, Colman I (2018). Global incidence of suicide among Indigenous peoples: a systematic review. BMC Med.

[3] Young TK, Revich B, Soininen L (2015). Suicide in circumpolar regions: an introduction and overview. Int J Circumpolar Health.

[4] Bjerregaard P (2001). Rapid socio-cultural change and health in the Arctic. Int J Circumpolar Health.

[5] Bjerregaard P, Larsen CVL (2016). Health aspects of colonization and the post-colonial period in Greenland 1721 to 2014. J North Stud.

[6] Bjerregaard P, Larsen CVL. Time trend by region of suicides and suicidal thoughts among Greenland Inuit. Int J Circumpolar Health. 2015;74:10.3402/ijch.v74.26053. 10.3402/ijch.v74.26053 PMC4336354 25701279

[7] Bjerregaard P, Larsen CVL (2018). Three lifestyle-related issues of major significance for public health among the Inuit in contemporary Greenland: a review of adverse childhood conditions, obesity, and smoking in a period of social transition. Public Health Rev.

[8] Pollock NJ, Apok C, Concepcion T, Delgado RA, Rasmus S, Chatwood S (2020). Global goals and suicide prevention in the Circumpolar North. Indian J Psychiatry.

[9] World Health Organization (2014). Preventing Suicide: A Global Imperative.

[10] Statistics Greenland. Statistics Greenland. Statistical Data Bank.: Statistics Greenland; 2022 [Available from: http://www.stat.gl.

[11] Bertelsen A (1935). Grønlandsk medicinsk Statistik og Nosografi. I. Grønlands Befolkningsstatistik 1901–30 (Greenland medical statistics and nosography. I. Population statistics 1901–30).

[12] Chief Medical Officer. Annual report. Nuuk Chief Medical Officer; 1951–1970.

[13] Ludwig B, Dwivedi Y (2018). The concept of violent suicide, its underlying trait and neurobiology: a critical perspective. Eur Neuropsychopharmacol.

[14] Cai Z, Junus A, Chang Q, Yip PSF (2022). The lethality of suicide methods: A systematic review and meta-analysis. J Affect Disord.

[15] Spein AR, Pedersen CP, Silviken AC, Melhus M, Kvernmo SE, Bjerregaard P (2013). Self-rated health among Greenlandic Inuit and Norwegian Sami adolescents: associated risk and protective correlates. Int J Circumpolar Health.

[16] Thorslund J (1990). Inuit suicides in Greenland. Arct Med Res.

[17] Bjerregaard P, Larsen CVL, Sørensen IK, Tolstrup JS (2020). Alcohol in Greenland 1950–2018: consumption, drinking patterns, and consequences. Int J Circumpolar Health.

[18] World Health O (2021). Live life: an implementation guide for suicide prevention in countries.

[19] Center for Suicide Research. Statistics 2022 [Available from: http://statistik.selvmordsforskning.dk/

[20] Iburg KM, Mikkelsen L, Richards N (2020). Assessment of the quality of cause-of-death data in Greenland, 2006–2015. Scand J Public Health.

[21] Thorslund J, Misfeldt J (1989). On suicide statistics. Arct Med Res.

[22] Statistics Denmark. Grønland. Folke- og boligtællingen 31. Copenhagen, Denmark. December 1970. (Greenland. Count of dwellings and census Dec. 31, 1970). Copenhagen: Copenhagen Statistics Denmark; 1974.

[23] Statistics Denmark (1978). Folke-ogboligtælling i Grønland 26. oktober 1976 (Count of dwellings and census in Greenland Oct. 26, 1976) [in Danish].

[24] Benchimol EI, Smeeth L, Guttmann A, Harron K, Moher D, Petersen I (2015). The REporting of studies Conducted using Observational Routinely-collected health Data (RECORD) statement. PLoS Med.

[25] StataCorp (2021). Stata Statistical Software: Release 17.

[26] Corporation M (2018). Microsoft Excel. Microsoft Corporation.

[27] World Health Organization. World Standard Population 2000–2025: World Health Organization; 2021 [Available from: https://seer.cancer.gov/stdpopulations/world.who.html.

[28] Wexler L, Silveira ML, Bertone-Johnson E (2012). Factors associated with Alaska Native fatal and nonfatal suicidal behaviors 2001–2009: trends and implications for prevention. Arch Suicide Res.

[29] Chachamovich E, Kirmayer LJ, Haggarty JM, Cargo M, McCormick R, Turecki G (2015). Suicide among Inuit: Results from a Large, Epidemiologically Representative Follow-Back Study in Nunavut. Can J Psychiatry.

[30] Sargeant H, Forsyth R, Pitman A (2018). The epidemiology of suicide in young men in Greenland: a systematic review. Int J Environ Res Public Health.

[31] Leineweber M, Arensman E (2003). Culture Change and Mental Health: The Epidemiology of Suicide in Greenland. Arch Suicide Res.

[32] Varnik A, Kolves K, van der Feltz-Cornelis CM, Marusic A, Oskarsson H, Palmer A (2008). Suicide methods in Europe: a gender-specific analysis of countries participating in the "European Alliance Against Depression". J Epidemiol Community Health.

[33] Dhungel B, Thapa A, Martínez-Rives NL, Takagi K, Martín P, Wada K (2022). Method-specific suicide trends from 1979 to 2016 among Japanese adolescents. J Affect Disord.

[34] Crawford A, Hicks J. Early childhood adversity as a key mechanism by which colonialism is mediated into suicidal behaviour. North Public Aff. 2018;5(3):18–22.

[35] Lindholm J, Pleisner JJ (2013). Psykologisk autoposistudie af de 7 selvmord i Tasiilaq i 2011 (Psychological autopsy study of the 7 suicides in Tasiilaq in 2011) [In Danish]. Nakorsanut.

[36] Grove O, Lynge J (1979). Suicide and attempted suicide in Greenland. A controlled study in Nuuk (Godthaab). Acta Psychiatr Scand..

[37] Leineweber M, Bjerregaard P, Baerveldt C, Voestermans P (2001). Suicide in a society in transition. Int J Circumpolar Health.

[38] Thorslund J (1991). Suicide among Inuit youth in Greenland 1977–86. Arctic Med Res.

[39] Ottendahl CB, Bjerregaard P, Svartá DL, Sørensen IK, Olesen I, Nielsen MS, et al. Mental sundhed og helbred blandt 15–34 årige i Grønland. Betydningen af opvækstvilkår, beskyttende faktorer og risikofaktorer (Mental health and health among 15–34 year olds in Greenland. The importance of childhood conditions, protective factors and risk factors.) [In Danish and Greenlandic]. Copenhagen, Denmark: National Institute of Public Health, Univerisy of Southern Denmark, Greenland CfPHi; 2021.

[40] Hicks J (2007). The social determinants of elevated rates of suicide among Inuit youth. Indigenous Affairs.

[41] Bombay A, Matheson K, Anisman H (2014). The intergenerational effects of Indian Residential Schools: implications for the concept of historical trauma. Transcult Psychiatry.

[42] Evans-Campbell T (2008). Historical trauma in American Indian/native Alaska communities: a multilevel framework for exploring impacts on individuals, families, and communities. J Interpers Violence.

[43] Wexler L (2013). Looking across three generations of Alaska Natives to explore how culture fosters indigenous resilience. Transcult Psychiatry.

[44] Redvers J, Bjerregaard P, Eriksen H, Fanian S, Healey G, Hiratsuka V (2015). A scoping review of Indigenous suicide prevention in circumpolar regions. Int J Circumpolar Health.

[45] MacDonald JP, Ford JD, Willox AC, Ross NA (2013). A review of protective factors and causal mechanisms that enhance the mental health of Indigenous Circumpolar youth. Int J Circumpolar Health.

[46] Ingemann C, Larsen CVL (2018). Well-being among indigenous children and youth in the Arctic–with a focus on Sami and Greenland Inuit: A scoping review. Copenhagen, Denmark: Nordic Council of Ministers, Nordic Council of Ministers Secretariat.

[47] Dahl-Petersen I, Larsen C, Nielsen N, Jørgensen M, Bjerregaard P (2016). Befolkningsundersøgelsen i Grønland 2014. Levevilkår, livsstil og helbred. (Population Health Survey in Greenland 2014. Living conditions, lifestyle, and health.) [In Danish and Greenlandic].

[48] Grønlands Politi (2018). Årsstatistik (Yearly statistics.) [In Danish].

[49] Rothman KJ, Greenland S, Lash TL (2008). Modern epidemiology: Lippincott Williams & Wilkin.

[50] Larsen CVL, Hansen CB, Ingemann C, Sørensen IK, Jørgensen ME, Olesen I (2019). Befolkningsundersøgelsen i Grønland 2018 - Levevilkår, livvstil og helbred (Population Health Survey in Greenland 2018. Living conditions, lifestyle, and health.) [In Danish and Greenlandic].

[51] Dube SR, Anda RF, Felitti VJ, Chapman DP, Williamson DF, Giles WH (2001). Childhood abuse, household dysfunction, and the risk of attempted suicide throughout the life span: findings from the Adverse Childhood Experiences Study. JAMA.

[52] Hall J, Porter L, Longhi D, Becker-Green J, Dreyfus S (2012). Reducing adverse childhood experiences (ACE) by building community capacity: a summary of Washington Family Policy Council research findings. J Prev Interv Community.

[53] Allen J, Rasmus SM, Fok CCT, Charles B, Henry D, Qungasvik T (2018). Multi-Level Cultural Intervention for the Prevention of Suicide and Alcohol Use Risk with Alaska Native Youth: a Nonrandomized Comparison of Treatment Intensity. Prev Sci.

[54] Barnett JD, Schmidt TC, Trainor B, Wexler L (2020). A pilot evaluation of culture camps to increase Alaska native youth wellness. Health Promot Pract.

[55] Cueva K, Rink E, Lavoie JG, Healey Akearok G, Guistini S, Kanayurak N (2022). From resilient to thriving: policy recommendations to support health and well-being in the arctic. Arctic.

[56] Pollock NJ, Healey GK, Jong M, Valcour JE, Mulay S (2018). Tracking progress in suicide prevention in Indigenous communities: a challenge for public health surveillance in Canada. BMC Public Health.

[57] Finlay S, Wenitong M (2020). Aboriginal community controlled health organisations are taking a leading role in COVID-19 health communication. Aust N Z J Public Health.

[58] Cueva K, Rink E, Lavoie JG, Stoor JPA, Healey Akearok G, Gladun E, et al. Diving below the surface: A framework for arctic health research to support thriving communities. Scand J Public Health. 2021;0(0):1–10. 10.1177/14034948211007694 33899601

[59] MIO. QAMANI [In Danish] (2022). Nuuk, Greenland National Advocacy Center working for Childrens Rights.

