# Correction: Time trends and geographical patterns in suicide among Greenland Inuit

**DOI:** 10.1186/s12888-023-04755-3

**Published:** 2023-04-13

**Authors:** Ivalu Katajavaara Seidler, Janne Schurmann Tolstrup, Peter Bjerregaard, Allison Crawford, Christina Viskum Lytken Larsen

**Affiliations:** 1 National Institute of Public Health, University of Southern Denmark, Copenhagen, Denmark; 2 Institute for Health and Nature, University of Greenland, Nuuk, Greenland; 3 Centre for Addiction and Mental Health, Toronto, Canada

**Correction: BMC Psychiatry 23, 187 (2023)**

**https://doi.org/10.1186/s12888-023-04675-2**

Following publication of the original article [1], the authors identified an error in Table 1. The correct Table 1 is given below.

**Table 1:** The five geographical regions used for stratification of suicide rates based on information from Statistics Greenland 2022 (10)

Region	Communities	Population size
Nuuk	The capital located in West Greenland	18,800 inhabitants in total
Central large communities in West Greenland	The largest communities in West Greenland, Maniitsoq and Sisimiut	8,108 inhabitants in total
Peripheral large communities in West Greenland	Larger communities often having more than 500 inhabitants in West Greenland	20,156 inhabitants in total
Small communities in West Greenland	Communities often having less than 500 inhabitants in West Greenland	6,179 inhabitants in total
East Greenland	All communities in East Greenland	3,110 inhabitants in total

The original article [1] has been corrected.
