# Supplementary material for: Time trends and geographical patterns in suicide among Greenland Inuit
Source: BMC Psychiatry. 2023 Mar 21;23:187. doi: 10.1186/s12888-023-04675-2 (PMC10031872; doi:10.1186/s12888-023-04675-2)
Supplement: Supplementary file 1 — Additional file 1. [file 12888_2023_4675_MOESM1_ESM.docx]

Appendix A

Table 1A. Suicide rates on five-year periods from 1970 to 2018 and the upper and lower 95% confidence intervals.

|  | Rate | 95% confidence interval | |
| --- | --- | --- | --- |
| Period |  | Lower | Upper |
| 1970-1974 | 28.7 | 21.2 | 36.2 |
| 1975-1979 | 46.9 | 37.5 | 56.4 |
| 1980-1984 | 100.0 | 86.4 | 113.5 |
| 1985-1989 | 120.5 | 106.1 | 135.0 |
| 1990-1994 | 101.6 | 88.8 | 114.5 |
| 1995-1999 | 102.5 | 89.8 | 115.1 |
| 2000-2004 | 94.7 | 82.7 | 106.8 |
| 2004-2009 | 83.7 | 72.4 | 95.0 |
| 2010-2014 | 88.2 | 76.6 | 99.8 |
| 2015-2018 | 81.3 | 68.8 | 93.8 |

Table 2A. Age specific suicide rates according to sex. Analysis of mortality data from 1970 to 2018.

|  | Men | 95% confidence interval | | Women | 95% confidence interval | |
| --- | --- | --- | --- | --- | --- | --- |
| Age | Rate | Low | High | Rate | Low | High |
| 10-14 | 21.7 | 13.2 | 30.2 | 14.5 | 7.4 | 21.6 |
| 15-19 | 255.3 | 225.2 | 285.5 | 95.9 | 77.0 | 114.8 |
| 20-24 | 386.8 | 348.6 | 425.1 | 90.4 | 71.5 | 109.3 |
| 25-29 | 254.5 | 222.3 | 286.6 | 78.3 | 59.9 | 96.6 |
| 30-34 | 158.8 | 132.5 | 185.1 | 55.7 | 39.6 | 71.8 |
| 35-39 | 149.2 | 122.9 | 175.6 | 36.8 | 23.2 | 50.4 |
| 40-44 | 133.1 | 107.4 | 158.8 | 45.2 | 29.5 | 60.8 |
| 45-49 | 91.7 | 69.4 | 114.0 | 31.9 | 18.3 | 45.6 |
| 50-54 | 98.9 | 73.6 | 124.1 | 34.4 | 18.9 | 49.9 |
| 55-59 | 73.3 | 48.7 | 97.9 | 27.5 | 12.0 | 43.1 |
| 60-64 | 58.2 | 32.7 | 83.7 | 20.5 | 5.3 | 35.6 |
| 65-69 | 41.4 | 15.8 | 67.1 | 22.9 | 4.6 | 41.3 |
| 70-74 | 46.3 | 12.0 | 80.5 | 16.1 | -2.1 | 34.2 |

Table 3A. Age specific suicide rates by 10-year birth cohorts. Analysis of mortality data from 1970 to 2018.

|  | 1940-1949 | | | 1950-1959 | | | 1960-1969 | | | 1970-1979 | | | 1980-1989 | | | 1990-1999 | | |
| --- | --- | --- | --- | --- | --- | --- | --- | --- | --- | --- | --- | --- | --- | --- | --- | --- | --- | --- |
| Age | Rate | Low | High | Rate | Low | High | Rate | Low | High | Rate | Low | High | Rate | Low | High | Rate | Low | High |
| 10-14 | 0 | 0.0 | 0.0 | 9.5 | -9.1 | 28.0 | 6.3 | 0.1 | 12.5 | 14.3 | 1.8 | 26.8 | 35.0 | 17.3 | 52.8 | 31.5 | 15.6 | 47.4 |
| 15-19 | 0 | 0.0 | 0.0 | 57.6 | 31.7 | 83.4 | 151.8 | 119.5 | 184.0 | 236.6 | 184.4 | 288.7 | 235.1 | 186.8 | 283.4 | 198.0 | 155.9 | 240.1 |
| 20-24 | 36.8 | -14.2 | 87.9 | 170.0 | 128.0 | 211.9 | 311.4 | 265.5 | 357.2 | 250.0 | 194.9 | 305.2 | 248.4 | 198.1 | 298.6 | 219.0 | 166.1 | 271.8 |
| 25-29 | 65.0 | 29.7 | 100.4 | 159.1 | 117.1 | 201.2 | 203.7 | 166.0 | 241.4 | 233.1 | 178.5 | 287.7 | 131.5 | 93.9 | 169.1 | 154.4 | 70.5 | 238.3 |
| 30-34 | 72.3 | 38.9 | 105.6 | 126.3 | 89.4 | 163.2 | 111.0 | 82.6 | 139.3 | 127.6 | 86.5 | 168.7 | 114.7 | 71.5 | 158.0 | 0.0 | 0.0 | 0.0 |
| 35-39 | 113.5 | 69.9 | 157.2 | 92.4 | 60.4 | 124.4 | 101.8 | 74.2 | 129.5 | 90.4 | 54.9 | 125.8 | 112.7 | 29.2 | 196.2 | 0.0 | 0.0 | 0.0 |
| 40-44 | 107.2 | 65.2 | 149.2 | 121.0 | 83.5 | 158.5 | 94.0 | 66.9 | 121.2 | 62.7 | 27.2 | 98.2 | 0 | 0.0 | 0.0 | 0.0 | 0.0 | 0.0 |
| 45-49 | 62.4 | 29.7 | 95.1 | 50.8 | 25.9 | 75.7 | 81.8 | 55.8 | 107.7 | 82.5 | 10.2 | 154.7 | 0 | 0.0 | 0.0 | 0.0 | 0.0 | 0.0 |
| 50-54 | 85.1 | 45.8 | 124.4 | 63.9 | 35.2 | 92.6 | 81.8 | 49.7 | 113.8 | 0.0 | 0.0 | 0.0 | 0 | 0.0 | 0.0 | 0.0 | 0.0 | 0.0 |
| 55-59 | 36.0 | 9.3 | 62.7 | 65.7 | 35.4 | 96.1 | 38.2 | -5.0 | 81.5 | 0.0 | 0.0 | 0.0 | 0 | 0.0 | 0.0 | 0.0 | 0.0 | 0.0 |

Table 4A. Age specific suicide rates by 10-year birth cohorts. Analysis of mortality data from 1970 to 2018.

|  | Nuuk | | | Central large communities in West Greenland | | | Peripheral large communities in West Greenland | | | Small communities in West Greenland | | | East Greenland | | |
| --- | --- | --- | --- | --- | --- | --- | --- | --- | --- | --- | --- | --- | --- | --- | --- |
| Period | Rate | Low | High | Rate | Low | High | Rate | Low | High | Rate | Low | High | Rate | Low | High |
| 1970-1974 | 56.3 | 28.5 | 28.5 | 30.7 | 21.3 | 21.3 | 20.2 | 9.6 | 9.6 | 17.9 | 12.4 | 12.4 | 52.5 | 38.9 | 38.9 |
| 1975-1979 | 74.7 | 31.2 | 31.2 | 35.5 | 22.0 | 22.0 | 45.6 | 14.1 | 14.1 | 23.5 | 14.6 | 14.6 | 93.0 | 50.6 | 50.6 |
| 1980-1984 | 201.4 | 48.6 | 48.6 | 42.6 | 23.1 | 23.1 | 94.2 | 20.0 | 20.0 | 41.1 | 19.6 | 19.6 | 198.6 | 72.3 | 72.3 |
| 1985-1989 | 109.1 | 33.0 | 33.0 | 58.1 | 26.1 | 26.1 | 150.4 | 24.7 | 24.7 | 79.2 | 27.4 | 27.4 | 210.6 | 73.0 | 73.0 |
| 1990-1994 | 77.3 | 26.0 | 26.0 | 79.7 | 29.5 | 29.5 | 107.0 | 20.4 | 20.4 | 71.9 | 25.7 | 25.7 | 266.0 | 80.4 | 80.4 |
| 1995-1999 | 83.7 | 25.6 | 25.6 | 63.3 | 25.9 | 25.9 | 108.0 | 20.3 | 20.3 | 66.6 | 24.7 | 24.7 | 303.0 | 84.8 | 84.8 |
| 2000-2004 | 65.8 | 21.8 | 21.8 | 57.1 | 24.4 | 24.4 | 94.7 | 18.9 | 18.9 | 97.8 | 30.3 | 30.3 | 269.4 | 79.6 | 79.6 |
| 2004-2009 | 53.4 | 18.8 | 18.8 | 69.1 | 26.5 | 26.5 | 82.3 | 17.7 | 17.7 | 83.5 | 28.9 | 28.9 | 235.0 | 73.8 | 73.8 |
| 2010-2014 | 55.1 | 18.3 | 18.3 | 55.3 | 23.6 | 23.6 | 93.9 | 19.1 | 19.1 | 89.9 | 31.7 | 31.7 | 249.7 | 76.4 | 76.4 |
| 2015-2018 | 36.1 | 15.8 | 15.8 | 53.4 | 26.2 | 26.2 | 93.6 | 21.6 | 21.6 | 82.5 | 35.3 | 35.3 | 245.3 | 86.3 | 86.3 |
